# Non-ST-Elevation Myocardial Infarction: A Heterogeneous Syndrome with Evolving Management—A Narrative Review

**DOI:** 10.3390/biomedicines14061379

**Published:** 2026-06-18

**Authors:** Silviu Raul Muste, Elena Emilia Babes, Cristiana Bustea, Luciana Dobjanschi, Francesca Andreea Muste, Dana Carmen Zaha

**Affiliations:** 1 Doctoral School of Biomedical Sciences, Faculty of Medicine and Pharmacy, University of Oradea, 410087 Oradea, Romania; muste.silviuraul@student.uoradea.ro (S.R.M.); eebabes@uoradea.ro (E.E.B.); furko.francescaandreea@student.uoradea.ro (F.A.M.); dzaha@uoradea.ro (D.C.Z.); 2 Department of Medical Disciplines, Faculty of Medicine and Pharmacy, University of Oradea, 410073 Oradea, Romania; 3 Department of Preclinical Disciplines, Faculty of Medicine and Pharmacy, University of Oradea, 410073 Oradea, Romania

**Keywords:** non-ST-segment elevation, multivessel coronary artery disease, complete coronary revascularization, percutaneous coronary intervention, elderly

## Abstract

Non-ST-segment elevation myocardial infarction (NSTEMI) has become the predominant form of acute coronary syndrome (ACS) and is frequently associated with multivessel coronary artery disease (MVD). Patients presenting with NSTEMI and MVD represent a particularly high-risk population characterized by advanced age, comorbidities, and an increased atherosclerotic burden. Although advances in pharmacological therapy and early invasive management have improved prognosis, the optimal revascularization strategy in this setting remains uncertain. In contrast to ST-segment elevation myocardial infarction (STEMI), where randomized controlled trials consistently support complete revascularization, evidence in NSTEMI with MVD is limited and is largely derived from observational studies and registry data. This has generated ongoing debate regarding whether complete revascularization offers superior outcomes compared with culprit-only percutaneous coronary intervention (PCI), and whether non-culprit lesions should be treated during the index procedure (immediate strategy) or in a staged manner. Current data suggest that complete PCI is generally associated with reduced recurrent ischemia, reinfarction, and repeat revascularization, with potential long-term survival benefits. However, patient comorbidities, lesion complexity, and procedural risk continue to influence outcomes, highlighting the importance of individualized decision-making. This narrative review synthesizes contemporary evidence on PCI-based revascularization strategies in NSTEMI with MVD, focusing on two central aspects: the extent of revascularization (complete versus incomplete) and the timing of intervention (single-stage versus staged). By integrating findings from registries, randomized trials and guideline recommendations, the review identifies areas of consensus, persisting uncertainties, and key evidence gaps. Ultimately, it underscores the need for large, dedicated trials to guide practice and optimize outcomes for NSTEMI patients with multivessel coronary disease.

## 1. Introduction

According to the Fourth Universal Definition of Myocardial Infarction [1], acute myocardial infarction (AMI) is defined by a rise and/or fall in cardiac troponin above the 99th percentile upper reference limit, together with evidence of myocardial ischemia such as symptoms, electrocardiographic changes, imaging abnormalities, or angiographic evidence of coronary thrombus. In contrast to ST-Segment Elevation Myocardial Infarction (STEMI), which typically results from an acute, complete coronary occlusion, non-ST-Segment elevation myocardial infarction (NSTEMI) is most often caused by subtotal or transient vessel obstruction related to plaque rupture, erosion, or endothelial dysfunction, frequently in the context of multivessel coronary artery disease (MVD).

NSTEMI has become the predominant manifestation of acute coronary syndrome (ACS) and represents a growing global health burden. Unlike other ACS entities, NSTEMI incidence has remained stable or even increased over recent decades, mainly due to changes in diagnostic criteria, population aging, and the widespread use of high-sensitivity cardiac troponins [2,3]. In the United States, more than one million people are hospitalized each year for myocardial infarction, and over 60% of these admissions are due to NSTEMI [4]. Despite advances in prevention and therapy, mortality remains a concern. For example, in the UK, the 180-day case fatality rate for NSTEMI decreased from 10.8% to 7.6% between 2003 and 2013, a trend also observed in the United States [5]. In Europe, the overall incidence of ACS is estimated at 215 per 100,000 annually, with NSTEMI showing a stable or rising trend compared to other forms of myocardial infarction [6,7]. The EuroHeart 2023 registry [8] confirmed the central role of NSTEMI, documenting 21,335 admissions across seven countries, surpassing other ACS subtypes. This reflects not only population aging but also improved survival after prior coronary events and more sensitive diagnostic tools. However, significant disparities persist; western European countries generally achieve lower ACS mortality and higher revascularization rates than eastern Europe. Romania continues to face one of the highest cardiovascular mortality rates in Europe, where cardiovascular disease accounts for approximately 55% of all deaths. These data highlight important regional inequalities in cardiovascular care and outcomes. The age-standardized mortality rate for acute myocardial infarction in 2017 was 104 per 100,000 men and 47 per 100,000 women. Data from the Romanian National Registry for non-ST-elevation acute coronary syndrome (NSTE-ACS) indicate that NSTEMI represents nearly one-third of all myocardial infarctions, while EuroHeart 2023 reported 1019 NSTEMI admissions in Romania between March and December 2022. Despite advances in treatment, mortality remains high, with a post-PCI rate of 6.56 per 100 patient-years in real-world settings [9].

Although NSTEMI is becoming increasingly prevalent relative to STEMI, data regarding the prevalence of MVD among NSTEMI patients remain unexpectedly scarce and contrasting with the comprehensive evidence available for STEMI and stable coronary artery disease (CAD) cohorts [10,11]. The clinical presentation of NSTEMI is heterogeneous and risk stratification is essential to guide therapeutic decisions [1].

Timely diagnosis and treatment, especially the modern ones [12], are crucial, as NSTEMI carries a substantial risk of recurrent ischemic events and mortality, particularly in high-risk subgroups such as the elderly, diabetics or those with chronic kidney disease [13,14]. Advancements in cardiac biomarkers, notably high-sensitivity cardiac troponins, have improved early detection and allowed for more precise patient triage [15]. Risk scores such as Global Registry of Acute Coronary Events (GRACE) or Thrombolysis in Myocardial Infarction (TIMI) facilitate identification of patients who benefit most from invasive strategies.

Therapeutic approaches in NSTEMI are broadly categorized into pharmacological and invasive strategies. Pharmacological therapy typically includes dual antiplatelet therapy (DAPT), anticoagulant, statins, beta-blockers and nitrates, initiated rapidly before and after revascularization depending on the extent of disease, clinical stability and patients’ choice. However, the optimal timing and completeness of revascularization in NSTEMI patients, especially those with MVD, remain controversial and a subject of ongoing investigation [16].

In this context, determining the optimal revascularization strategy represents a central challenge in the management of NSTEMI patients with multivessel coronary artery disease. Current practice varies widely between centers and operators, reflecting differences in patient risk profiles, angiographic complexity, and the availability of advanced physiological or imaging tools. The key questions revolve around whether revascularization should target only the culprit lesion or aim for complete treatment of all significant stenoses, and whether additional lesions should be addressed during the index procedure or staged at a later time. These decisions have important implications for both short- and long-term outcomes, including recurrent ischemia, heart failure progression, repeat hospitalization, and survival [17]. As such, understanding the evidence behind each strategy is essential to guiding individualized, patient-centered care.

This narrative review aims to highlight revascularization strategies in patients with NSTEMI and multivessel coronary artery disease undergoing percutaneous coronary intervention, with a focus not only on the choice between complete and incomplete revascularization but also on the optimal timing of intervention.

## 2. Methods

**Figure 1:**
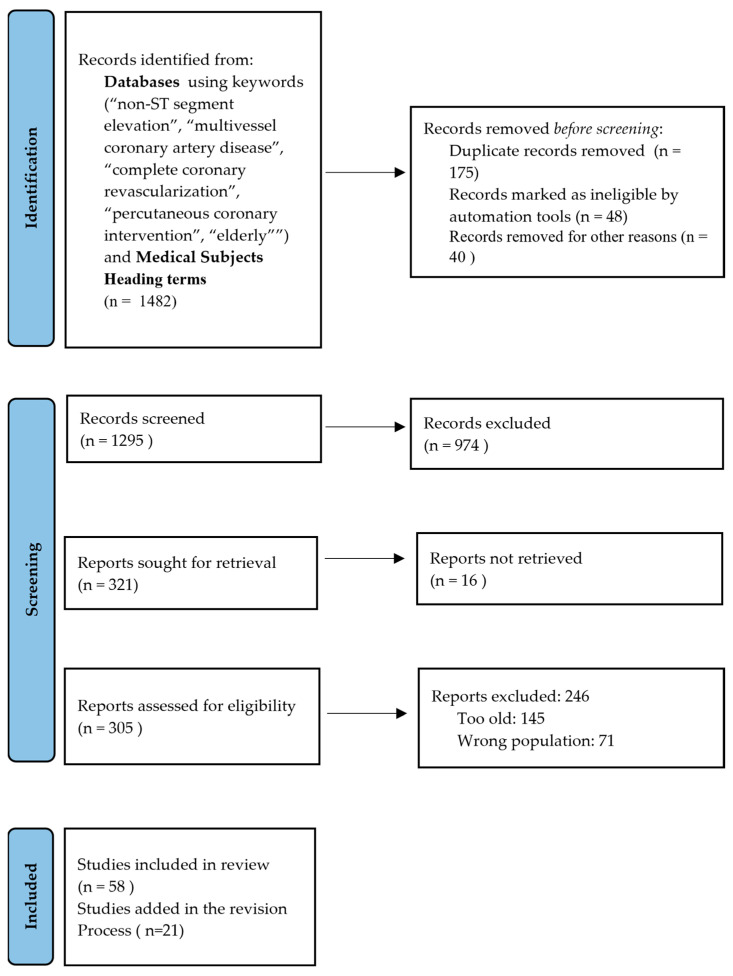
PRISMA flow chart.

A comprehensive literature search was conducted using the PubMed and Google Scholar databases to identify full-text original research articles and review papers. The timeframe for inclusion was limited to the past ten years (2016–2025). The search strategy included combinations of the following keywords: “non-ST segment elevation”,” multivessel coronary artery disease”, “complete coronary revascularization”, “percutaneous coronary intervention”, “elderly”. Medical Subject Heading (MeSH) was also used for including the most relevant studies. Although this is not a systematic review, a PRISMA flow diagram was used to illustrate the selection of included studies as shown in Figure 1 [18]. After the initial search, the retrieved articles were screened, and duplicate records were excluded to ensure the uniqueness of the selected studies.

## 3. Pathophysiological Considerations

Myocardial infarction (MI) is pathologically characterized by the irreversible death of cardiac myocytes following a sustained period of inadequate blood supply.

**Figure 2:**
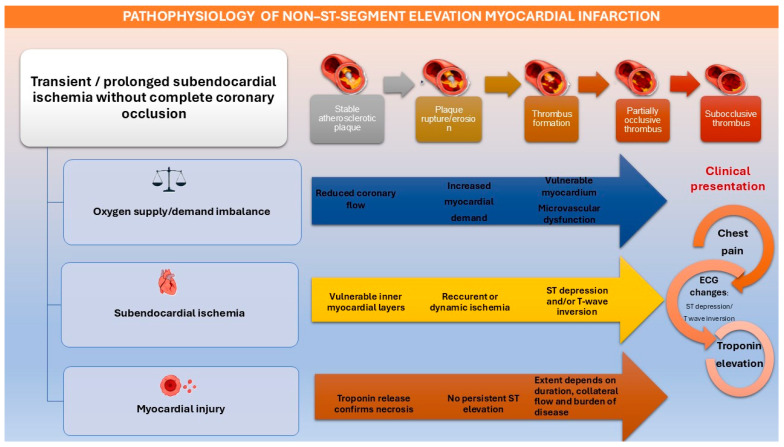
Pathophysiology of Non-ST-segment Elevation Myocardial Infarction.

NSTEMI arises from a heterogeneous spectrum of pathophysiological mechanisms, differing from the complete coronary occlusion observed in STEMI. In most cases, the underlying cause in NSTEMI is a partial or transient obstruction of a coronary artery, leading to subendocardial ischemia without transmural myocardial injury as shown in Figure 2. However, a proportion of patients experiencing total coronary occlusion may present with a clinical picture consistent with NSTEMI, lacking the typical ST-segment elevation on electrocardiogram [19]. The meta-analysis by Abdur R Khan et al. [20], which included 40.777 patients with NSTEMI, demonstrated that approximately 25.5% had an acutely total occluded culprit coronary artery, and this atypical presentation was associated with increased mortality and major adverse cardiac events (MACE). Similarly, a more recent analysis from the CATHPCI National Cardiovascular Registry by Kherallah et al. [21], which included STEMI and NSTEMI patients, in whom PCI was performed between 2018–2023, showed that, although only approximately 15% of NSTEMI patients presented acute total coronary occlusion, they were at a significantly greater risk of in-hospital MACE as compared with NSTEMI without acute total coronary occlusion. These findings highlight the potential for under-recognition and delayed treatment in this subgroup.

Beyond partially occlusive coronary lesions, several additional mechanisms may contribute to NSTEMI by disrupting the balance between myocardial oxygen supply and demand. These include coronary vasospasm (e.g., Prinzmetal angina), coronary embolism, inflammatory involvement of the coronary vasculature (such as arteritis), myocarditis, and exposure to cardiotoxic agents. Furthermore, systemic conditions (including severe hypotension, uncontrolled hypertension, tachyarrhythmias, aortic stenosis, and pulmonary embolism) may precipitate NSTEMI even in the absence of significant obstructive coronary artery disease, by increasing myocardial oxygen demand or reducing perfusion [22].

Patients presenting with NSTEMI are typically older and more frail and have a higher burden of comorbidities. Multivessel disease is present in a substantial proportion of cases and reflects a complex interplay between systemic atherosclerosis, inflammation, and microvascular dysfunction, contributing to worse outcomes compared with single-vessel disease. Patients with MVD (defined as ≥70% stenosis in ≥2 major epicardial vessels) have a more extensive and unstable atherosclerotic burden. Angiographic and intracoronary imaging studies have shown that non-culprit plaques often possess high-risk features such as thin fibrous caps, large lipid cores, and active inflammation. The PROSPECT II study [23], a multicentric prospective study, demonstrated that approximately half of MACE in ACS patients originates from non-culprit lesions with vulnerable morphologies. Patients with MVD have significantly higher total ischemic burden, even when only the culprit lesion is treated. This cumulative effect explains the higher incidence of heart failure and mortality in NSTEMI patients with untreated non-culprit lesion [24]. These findings emphasize that NSTEMI is often a systemic disease with multifocal vulnerability rather than a single-lesion event.

## 4. Treatment

A crucial component in the care of NSTEMI patients is evidence-based management. This includes immediate pharmacologic stabilization, early risk-adapted decisions regarding coronary angiography and long-term secondary prevention. The therapeutic approach must be individualized based on ischemic and bleeding risk, hemodynamic status, extent of coronary artery disease, comorbidities and patient preference.

### 4.1. Initial Risk Stratification in NSTEMI

Because therapeutic decision-making in NSTEMI relies on distinguishing patients who will benefit from an early invasive strategy from those suitable for a more conservative approach, early and accurate risk stratification is fundamental. Contemporary guidelines emphasize that clinical impression alone is often insufficient in evaluating ischemic risk; therefore, structured risk scores integrating clinical history, ECG changes, biomarker levels, and hemodynamic parameters are indispensable in guiding management pathways. Within the emergency department setting, several validated tools—most prominently the GRACE, TIMI and HEART (History, ECG, Age, Risk factors, Troponin) scores, are routinely employed to estimate the likelihood of major adverse cardiovascular events in patients presenting with suspected NSTEMI [25].

Among these, the GRACE risk score occupies a central and guideline-endorsed position in NSTEMI, being recommended by both the European Society of Cardiology (ESC) and the American Heart Association/American College of Cardiology (AHA/ACC) as the preferred tool for predicting in-hospital and 6-month mortality and for determining the urgency of invasive management. A GRACE score >140 identifies NSTEMI patients at high ischemic risk who derive significant benefit from early coronary angiography (within 24 h), whereas lower scores allow for a more delayed or selective invasive approach. Although initially derived from a heterogeneous ACS cohort, the model has been consistently validated in dedicated NSTEMI populations, demonstrating superior prognostic accuracy compared to physician assessment alone and helping overcome the well-recognized risk–treatment paradox, in which high-risk patients often receive disproportionately conservative care [26,27,28]. It provides an objective and reproducible estimate of risk, effectively overcoming subjective clinical biases and mitigating the well-established risk–treatment paradox, whereby high-risk patients paradoxically receive less guideline-directed therapy. Its applicability is facilitated by the availability of electronic calculators and by the model’s reliance on universally obtainable variables such as age, hemodynamics, renal function, biomarkers, ECG findings, and Killip class. Multiple iterations of the score (GRACE 1.0 and GRACE 2.0) have been developed, maintaining similar clinical parameters while adjusting weightings and improving usability; GRACE 2.0 additionally allows validated substitutions when specific data are missing [29,30,31]. Beyond guiding decisions regarding the timing of invasive management, risk scores also influence decisions about the appropriate level of monitoring and resource allocation in NSTEMI. Recent discussions have reconsidered the routine admission of all NSTE-ACS patients to intensive care units, emphasizing instead a risk-stratified approach. Savonitto and Morici [32] argue that ICU admission should be reserved for patients identified as high risk based on structured assessments, including clinical presentation, ECG abnormalities, biomarker elevation, and laboratory parameters, while noting that the GRACE score, although a robust prognostic tool, does not account for operator, procedural, or institutional factors. Thus, selective ICU allocation guided by individualized risk estimation is both safer and more efficient. Additionally, the role of GRACE as a triage instrument has been questioned by findings from the UKGRIS (UK GRACE Risk Intervention Study) [33]. This large cluster-randomized trial of more than 3000 NSTE-ACS patients demonstrated no significant differences in guideline adherence or clinical outcomes (such as cardiovascular death, myocardial infarction, or hospital readmission) between GRACE-guided management and standard care. These results suggest that in healthcare systems already delivering high-quality ACS management, the incremental benefit of mandatory GRACE-based triage may be limited. Even so, the GRACE score continues to serve as a valuable prognostic and risk-stratification tool, particularly in settings characterized by treatment variability or a persistent risk–treatment paradox.

The TIMI risk score remains another widely used tool for the early assessment of NSTEMI. Developed originally for patients with unstable angina/NSTE-ACS, the TIMI score employs a simple 0–7 scale based on variables readily available at presentation (such as age ≥65, known coronary artery disease, multiple risk factors, ST-segment deviation, elevated biomarkers, aspirin use, and recent angina) to estimate the short-term risk of death, myocardial infarction, or urgent revascularization. Although less granular and slightly less accurate than GRACE in mortality prediction, TIMI is valued for its simplicity, speed, and utility in acute care settings for early triage towards invasive strategies [34,35,36].

The HEART score, although primarily used as a chest-pain stratification tool in emergency departments, may complement NSTEMI evaluation by distinguishing low-risk patients suitable for early discharge from those warranting closer monitoring or expedited diagnostic testing [37]. By combining objective variables with clinical judgment, it refines early risk categorization and supports decision-making.

Beyond predicting short-term ischemic events, clinical risk scores have also been investigated and tested as surrogate markers of coronary lesion burden in NSTE-ACS/NSTEMI. Among them, the HEART score consistently shows the strongest association with angiographic complexity, with higher HEART values correlating with higher SYNTAX (Synergy Between PCI With Taxus And Cardiac Surgery) scores and multivessel or complex disease, and outperforming both GRACE and TIMI in most cohorts. GRACE demonstrates a moderate but significant correlation with SYNTAX and may help to flag patients with extensive coronary artery disease, although its discriminatory ability for high SYNTAX categories is generally lower than that of HEART. In contrast, TIMI shows only modest correlations with angiographic severity and appears to be the least accurate of the three for predicting anatomical complexity, retaining its primary role as a simple predictor of short-term ischemic risk rather than a reliable surrogate of coronary lesion extension [38,39].

Importantly, the role of risk stratification differs fundamentally between NSTEMI and STEMI [40]. In STEMI, the imperative for immediate reperfusion therapy renders risk scores primarily prognostic. In contrast, in NSTEMI, risk scores directly inform decisions regarding the timing and intensity of invasive management, underscoring their central role within modern NSTEMI treatment algorithms.

### 4.2. Percutaneous Coronary Intervention in NSTEMI

#### 4.2.1. Up to Date—Current Guidelines’ Recommendations

**Figure 3:**
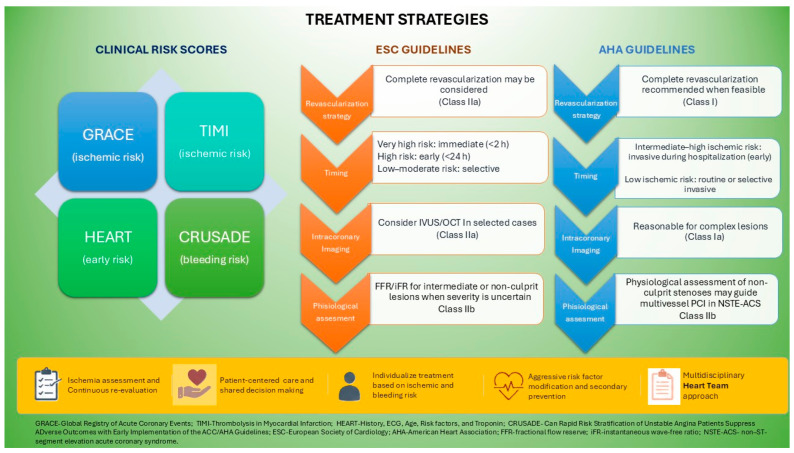
Treatment strategies.

Compared with STEMI, myocardial injury in NSTEMI is generally less extensive, but the long-term prognosis may be worse, especially in elderly patients, diabetics, and those with MVD. Early revascularization is therefore a cornerstone of management, aimed at limiting ischemia, preventing recurrent events, and improving outcomes. Both the ESC 2023 [16] and AHA 2025 [41] guidelines emphasize the role of early invasive evaluation, but they differ in definitions of timing and risk stratification as shown in Figure 3.

The ESC 2023 guideline recommends an immediate invasive strategy (i.e., emergency angiography with PCI if indicated) in very high-risk patients such as those with cardiogenic shock, refractory angina, acute heart failure from ischemia, or life-threatening arrhythmias. In high-risk patients (e.g., GRACE score >140, dynamic ECG changes), there is a class I recommendation for an inpatient invasive approach, with a class IIa recommendation for angiography within 24 h. Stable, non-high-risk patients may undergo either inpatient or selective invasive strategies, with coronary angiography guided by non-invasive testing. Evidence shows that an early invasive approach lowers recurrent ischemia and hospital stay but does not consistently reduce all-cause mortality. The AHA 2025 guideline stratifies patients into very high, high, intermediate, and low risk categories. Immediate angiography within 2 h is recommended for very high-risk patients, while routine angiography within 24–72 h is indicated for high or intermediate risk. For low-risk patients, selective invasive strategies or non-invasive confirmation with coronary computed tomography angiography are appropriate.

Management of NSTEMI with MVD remains a critical area of uncertainty. Unlike STEMI, where complete revascularization during the index PCI or within 45 days is class I, level A [16], evidence in NSTEMI is limited. The ESC 2023 guideline provides only a class IIa, level C recommendation for complete revascularization during the index procedure, while the AHA 2025 guideline assigns a stronger class I, level B-R recommendation for PCI of significant non-culprit lesions, either during the index procedure or as a staged strategy. These discrepancies highlight the lack of dedicated randomized trials exclusively in NSTEMI with MVD, with most data extrapolated from STEMI or mixed ACS cohorts.

In daily practice, this uncertainty is further complicated by the limitations of angiography alone, which may not reliably distinguish functionally relevant non-culprit stenoses from anatomically significant but hemodynamically non-significant lesions, nor adequately characterize lesion morphology in complex coronary anatomy. Therefore, adjunctive lesion assessment using invasive coronary physiology and intracoronary imaging has become increasingly important for tailoring the extent and technical optimization of revascularization in NSTEMI patients with multivessel disease. Regarding invasive coronary physiology, the 2023 ESC Guidelines state that, in hemodynamically stable patients with NSTE-ACS and multivessel disease undergoing PCI, functional invasive evaluation of non-infarct-related artery stenosis severity during the index procedure may be considered to guide the decision for complete revascularization (class IIb, level B). However, the ESC guidelines also emphasize that PCI of the infarct-related artery should not be deferred solely on the basis of invasive epicardial functional assessment in the acute ACS setting. Similarly, the 2025 AHA Guideline states that, in patients with NSTE-ACS in whom multivessel PCI is being considered, physiological assessment of a non-culprit stenosis may be considered to guide revascularization decisions (class IIb, level B-R). In contrast, intracoronary imaging has a complementary role, primarily supporting culprit-lesion identification, plaque characterization, lesion preparation, stent sizing, and PCI optimization rather than determining hemodynamic significance. The 2023 ESC Guidelines highlight the usefulness of IVUS or OCT in ACS patients when angiography is inconclusive, particularly in cases without significant obstructive coronary artery disease, ambiguous culprit lesions, suspected plaque rupture or erosion, spontaneous coronary artery dissection, or complex PCI. The 2025 AHA Guideline provides a stronger recommendation for procedural guidance, stating that in ACS patients undergoing coronary stent implantation in the left main artery or in complex lesions, intracoronary imaging with IVUS or OCT is recommended to guide PCI and reduce ischemic events (class I, level A). Thus, both contemporary guidelines support an individualized, anatomy-, physiology-, and imaging-guided approach to revascularization in NSTEMI patients with multivessel disease, although physiological assessment of non-culprit lesions remains a weaker recommendation than intracoronary imaging-guided optimization of complex PCI. Recent evidence further supports the clinical relevance of this approach. In an analysis from the Polish Registry of Acute Coronary Syndromes [42], the use of IVUS and FFR in invasively treated ACS patients was associated with procedural and prognostic implications, emphasizing the growing importance of intracoronary assessment in contemporary ACS management. Moreover, the iLITRO-EPIC07 study [43] showed that, in patients with intermediate left main coronary artery stenosis, a hybrid decision-making strategy based on iFR, FFR, and IVUS was feasible, with IVUS being particularly useful in cases of discordant physiological results and deferral of revascularization appearing safe when guided by this integrated approach. Therefore, in NSTEMI patients with MVD, FFR, iFR, and IVUS should be regarded as complementary tools that may help identify functionally significant non-culprit lesions, avoid unnecessary PCI, optimize stent implantation, and support a more individualized revascularization strategy.

Current evidence supports the role of early invasive management in moderate-to-high-risk NSTEMI patients, but optimal timing and the extent of revascularization in MVD remain unresolved. In particular, the absence of randomized controlled trials focused solely on NSTEMI with MVD means that guideline recommendations are still based on lower levels of evidence. Future studies are needed to clarify whether immediate complete revascularization, staged approaches, or culprit-only strategies provide the best long-term outcomes in this high-risk population.

#### 4.2.2. Does Revascularization Strategy Matter in NSTEMI? The Interplay Between Complete vs. Incomplete PCI and Optimal Timing

First and foremost, it is crucial to emphasize the clinical impact and potential advantages of complete revascularization compared with incomplete revascularization, as this distinction represents a central question in the management of NSTEMI patients with multivessel coronary artery disease.

In patients with NSTEMI, a substantial proportion of recurrent ischemic events arise from non-culprit coronary arteries, not just from the originally treated culprit lesion. Many of these non-culprit events occur later in follow-up, underscoring the diffuse and progressive nature of coronary artery disease. Culprit-only revascularization may leave patients vulnerable to future events from untreated lesions and provides a strong pathophysiological rationale for considering more complete revascularization strategies in NSTEMI patients with MVD [24,44].

Thus, the pathophysiological rationale for complete revascularization is directly linked to the clinical problem of residual, untreated atherosclerotic burden, which provides the framework for interpreting the outcome studies summarized below.Understanding the balance between these two strategies provides the foundation for evaluating not only short-term safety but also long-term outcomes such as recurrent ischemia, reinfarction, and survival. Over the past decade, a growing body of observational evidence has explored the prognostic implications of revascularization strategy in patients presenting with NSTEMI and MVD. Although randomized trial data in this population remain limited, several registries and cohort studies have consistently highlighted potential advantages of complete revascularization over culprit-only PCI.

To improve readability, the available evidence is discussed in a stepwise manner: first, studies comparing complete and culprit-only revascularization are presented; second, sources of heterogeneity and conflicting results are considered; and finally, the evidence is synthesized through meta-analyses.

A large multicenter registry, the BleeMACS (Bleeding Complications in a Multicenter Registry of Patients Discharged with a Diagnosis of Acute Coronary Syndrome) [45], evaluated 4520 patients with myocardial infarction and multivessel coronary artery disease, of whom approximately one-third presented with NSTEMI. In this cohort, complete revascularization (CR) was achieved in about 42% of NSTEMI patients, who generally exhibited a greater angiographic disease burden, including more frequent three-vessel disease and the need for multiple stents. Among NSTEMI patients, CR was consistently associated with improved clinical outcomes. At one-year follow-up, CR resulted in significantly lower rates of all-cause mortality (4.5% vs. 8.5%), reinfarction (3.7% vs. 6.6%), and overall MACE (8.1% vs. 13.9%) compared with incomplete revascularization (IR). These benefits remained robust after propensity score matching, underscoring that the advantage of CR was not solely attributable to baseline differences between groups. The registry therefore supports the concept that, in NSTEMI patients with multivessel disease, complete revascularization is both safe and associated with a meaningful reduction in adverse cardiovascular events. The benefit of CR regarding MACE and mortality at long term follow up is also sustained in numerous studies which included patients with acute coronary syndromes and multivessel disease [46,47].

Rathod et al. [48] conducted a large, multicenter observational study to assess the impact of single-stage complete revascularization versus culprit-only (CO) by PCI in patients presenting with NSTE-ACS and MVD. The study analyzed a data from 21.857 NSTEMI and MVD patients, drawn from a registry of over 37.000 patients treated across eight heart attack centers in London between 2005–2015, over a median follow-up of 4.6 years. Approximately 53.7% of patients underwent CR during the index procedure, while the remainder received CO-PCI. Patients undergoing CR were older and more likely to be male, diabetic, or have renal disease and a history of previous MI or revascularization. In the complete group, more vessels were treated per procedure, with longer total stent length; left anterior descending (LAD) and left main (LM) interventions were more frequent, whereas CO-PCI was more common in the right coronary artery (RCA). In-hospital composite MACE was similar (4.1% CR vs. 3.8% CO), though in-hospital death was higher with CR (2.3% vs. 1.5%), likely reflecting greater baseline complexity; repeat in-hospital PCI was lower with CR. Over follow-up, Kaplan–Meier curves showed lower mortality with CR (22.5% vs. 25.9%; log-rank *p* = 0.0005). After adjustment, CR remained associated with improved survival (multivariable hazard ratio (HR) 0.90, 95% confidence interval (CI) 0.85–0.97) and in a 1:1 propensity-matched cohort (HR 0.89, 95% CI 0.76–0.98). Landmark analyses suggested the long-term advantage of CR emerges after 6 months, once early in-hospital risk differences abate. CR in one setting was associated with better long-term survival than treating only the culprit lesion, despite higher early procedural risk, supporting completeness of revascularization.

A single-center retrospective cohort study [49] evaluated 202 patients with NSTE-ACS and MVD treated between 2010 and 2013 at a PCI-capable center in Portugal. Of these, 35.1% underwent multivessel revascularization, whereas 64.9% received CO-PCI. Propensity-score matching generated two comparable groups of 66 patients each, enabling balanced long-term outcome assessment over a mean follow-up of approximately 4.2 years. Procedurally, multivessel treatment was performed in one setting in 66.2% and staged within 30 days in 32.4%; complete revascularization was achieved in 52.1% of multivessel cases. When incomplete, the main barriers were diffuse/small-vessel disease and chronic total occlusions (CTO), and decisions to defer non-culprit PCI were often driven by moderate (<70%) stenoses (35.1%). In the CO group, the most frequently treated arteries were the left circumflex artery (LCx) (35.9%) and LAD (35.1%), and 35.9% of patients had >1 untreated artery. In-hospital adverse events were similar between strategies after matching. On longer-term follow-up (matched cohorts), multivessel revascularization was associated with lower rates of: reinfarction (4.5% vs. 16.7%, log-rank *p* = 0.018), unplanned revascularization (6.1% vs. 16.7%, *p* = 0.048), unplanned PCI (3.0% vs. 13.6%, *p* = 0.023), and the composite of death/reinfarction/unplanned revascularization (16.7% vs. 31.8%, *p* = 0.046) compared with culprit-only PCI.

These early observational data therefore point in a similar direction, suggesting that more complete treatment of significant coronary lesions may reduce recurrent ischemic events; however, the following studies also show that this benefit is not uniform across all populations or endpoints. Although these studies reported no difference in in-hospital mortality between complete and incomplete PCI, not all datasets align with this neutral signal. A hospital-based observational study by Iqbal et al. [50] found that in-hospital deaths clustered in the incompletely revascularized group, suggesting a short-term hazard from leaving significant non-culprit disease untreated. Another study by Carvalho et al. [51] examined complete revascularization in NSTEMI patients with MVD. The investigators assessed both in-hospital outcomes (overall complications and mortality), and 1-year endpoints including all-cause mortality and unplanned cardiovascular rehospitalization. In-hospital complication rates were similar between groups. However, there was a trend toward lower in-hospital mortality with complete revascularization (0.7% vs. 1.6%; *p* = 0.06). At 1 year, complete revascularization was associated with significantly fewer cardiovascular rehospitalizations (9.3% vs. 18.8%; *p* < 0.001). Mortality at 1 year remained comparable, indicating a neutral effect on long-term survival.

A large single-center study from China [52] evaluated 3338 NSTE-ACS patients with multivessel CAD undergoing PCI in 2013. CO-PCI was performed in 2259 patients, and multivessel PCI (MV-PCI) in 1079. At 2 years, there was no significant difference in Major Adverse Cardiac and Cerebrovascular Events (MACCE) (13.1% vs. 14.0%) between the groups after multivariable adjustment and propensity analyses. Subgroup analyses were consistent across patient categories. This study found no clear advantage of MV-PCI over CO-PCI in NSTEMI. This divergence highlights important nuances: whereas most registries suggest that treating all significant lesions improves long-term outcomes, Li et al. raise the possibility that in some populations or practice settings, the additional procedural complexity of complete revascularization may not translate into incremental clinical benefit. These conflicting findings emphasize that baseline risk, angiographic complexity, and procedural strategies may critically shape the impact of complete PCI in NSTEMI.

Also, in a comprehensive analysis from the Netherlands Heart Registration, Pustjens et al. [53] investigated the comparative effectiveness of MV-PCI versus CO-PCI among 10,507 patients presenting with NSTE-ACS and angiographically confirmed multivessel coronary artery disease. After rigorous adjustment through multiple imputation, propensity score matching, and Cox regression, long-term all-cause mortality remained comparable between strategies (10.7% vs. 10.2% in the unmatched cohort; 9.7% vs. 11.0% post-matching), with no differences observed for one-year mortality or periprocedural reinfarction. By contrast, MV-PCI conferred a substantial reduction in subsequent coronary reinterventions (10.6% vs. 18.1% post-matching, *p* < 0.001), along with lower rates of urgent coronary artery bypass grafting (CABG) and target vessel revascularization at one year. Subgroup analyses revealed a non-significant trend toward mortality reduction among women and non-diabetic patients, whereas the reintervention benefit of MV-PCI was consistent across clinical strata. The authors concluded that, in the setting of NSTE-ACS and multivessel disease, complete revascularization primarily improves event-free survival through a reduction in repeat procedures, without translating into a demonstrable mortality advantage during the median follow-up of approximately two years. Nonetheless, interpretation is constrained by the observational design, reliance on angiographic rather than physiological lesion assessment, limited detail on disease complexity.

In a prospective observational study, Pandit et al. [54] enrolled 60 patients with NSTEMI and multivessel coronary disease, divided equally between target-vessel revascularization (*n* = 30) and CR (*n* = 30). At six months of follow-up, the primary composite outcome of all-cause death, nonfatal myocardial infarction, or ischemia-driven revascularization did not differ significantly between the two strategies. However, important differences emerged in secondary endpoints: patients treated with CO-PCI experienced more frequent hospitalizations for heart failure (6 vs. 1 at 1 month; 7 vs. 2 at 6 months, *p* < 0.05) and higher rates of recurrent angina (8 vs. 2 at 1 month; 9 vs. 3 at 6 months, *p* < 0.05) compared to those undergoing CR. Thus, although the trial did not demonstrate a short-term survival or infarction benefit, it highlighted that complete revascularization may confer superior symptomatic relief and reduced heart failure burden, underscoring patient-centered advantages even in the absence of a clear mortality effect.

In the recent e-ULTIMASTER registry sub-analysis [55], which included 3832 patients presenting with NSTEMI and multivessel disease, CR was achieved in 47% of cases, while 53% remained IR. Patients in the CR group were younger and had fewer comorbidities (diabetes, hypertension, renal impairment, prior MI/PCI/CABG), but underwent more extensive interventions, with a greater mean number of lesions treated (1.9 vs. 1.4), more stents implanted (2.2 vs. 1.6), and longer cumulative stent length (38.7 mm vs. 31.7 mm, all *p* < 0.0001). CR was also more frequently performed in the right coronary artery (45.3% vs. 31.7%), left anterior descending (58.6% vs. 45.8%), and left circumflex (53.1% vs. 36.4%), while the prevalence of left main PCI was similar between groups (≈5%). At one year, CR was associated with a significantly lower rate of the patient-oriented composite endpoint (POCE: all-cause death, any myocardial infarction, and any repeat revascularization) compared with IR (7.0% vs. 12.9%; adjusted 7.7% vs. 12.0%, *p* < 0.0001). This benefit was largely driven by reductions in all-cause mortality (2.7% vs. 4.2%, *p* = 0.02) and repeat revascularizations (4.9% vs. 7.9%, *p* < 0.001), particularly non-target vessel revascularizations. Although unadjusted analyses suggested fewer target lesion failures (3.6% vs. 5.5%, *p* < 0.01), this difference lost statistical significance after adjustment. Overall, the study supports the prognostic value of achieving CR during the index hospitalization in NSTEMI patients with multivessel disease.

The variability among these registries indicates that the effect of complete revascularization should not be interpreted in isolation. Patient selection, anatomical complexity, procedural risk, and the quality of PCI all modify the observed relationship between revascularization completeness and clinical outcome. Other studies evaluated the efficacy of complete revascularization based on the generation of stent used.

This technical perspective helps explain why some studies show a stronger benefit of complete revascularization than others, particularly in the context of contemporary drug-eluting stents and improved procedural optimization.

Kim et al. [56] analyzed data from the Korea Acute Myocardial Infarction Registry (KAMIR), focusing on 4588 patients with NSTEMI and MVD who underwent PCI with newer-generation drug-eluting stents (DES). Patients were grouped into CO-PCI (*n* = 2055) versus MV-PCI (*n* = 2533); within the latter, CR (*n* = 2029) and IR (*n* = 504) were distinguished. The follow-up was 2 years, and the primary endpoint was MACE; secondary endpoints included stent thrombosis. Patients undergoing CR were younger, with better renal function, lower N-terminal pro-B-type Natriuretic Peptide, and higher left ventricular ejection fraction (LVEF) compared with IR. Angiographically, LAD was the most frequent culprit vessel, but CR involved treatment of more non-culprit vessels (LAD, LCx, RCA), while IR patients more often had residual three-vessel disease (≈60% vs. 36% in CR). Lesion complexity (type C, bifurcations, CTOs) was higher in IR and CO-PCI groups, whereas CR required longer stent lengths and more extensive PCI. At 2-year follow-up, the incidence of MACE and stent thrombosis was similar across strategies after adjustment. However, non-target vessel revascularization occurred significantly more often after CO-PCI and IR compared with CR or MV-PCI, underscoring the protective effect of treating nonculprit lesions. The study indicates that, although complete revascularization may not confer a significant reduction in major adverse cardiovascular endpoints over the medium term, it is associated with a decreased need for subsequent revascularization and mitigates the risk related to residual coronary lesions. In the study by Hsieh et al. [57], 702 NSTEMI patients with MVD, who underwent PCI with either first-generation or second-generation drug-eluting stents were analyzed to compare long-term outcomes between complete revascularization and incomplete revascularization. It was observed that the benefit of CR was only apparent with second-generation DES; with older stents, CR offered no clear advantage over IR in terms of MACE. The data also suggest that leaving untreated disease is especially harmful when stent performance is optimal (as with second generation DES), likely because residual lesions become sources of later ischemic events.

Several recent systematic reviews and meta-analyses have consistently demonstrated that, among patients with NSTEMI and multivessel coronary artery disease, complete revascularization is associated with superior clinical outcomes compared with a culprit-only strategy, especially on long term follow-up, supporting its role as the preferred approach whenever anatomically and clinically feasible.

A meta-analysis of 17 observational studies [58] comprising ~160,341 NSTEMI patients with multivessel disease compared MV-PCI and CO-PCI. In the short term, no statistically significant differences were found in mortality (RR 0.917, 95% CI 0.613–1.371) or MI (RR 0.960, 95% CI 0.612–1.507). However, over longer follow-up, MV-PCI was associated with significantly lower long-term all-cause mortality (RR 0.746, 95% CI 0.644–0.865) and reduced repeat revascularization (RR 0.775, 95% CI 0.629–0.954). The authors conclude that while early outcomes are similar, MV-PCI may offer superior long-term benefit.

Similarly, Prajapati and colleagues [59] conducted a systematic review and meta-analysis which included nearly 37,000 patients with NSTE-ACS and MVD, comparing CR with a CO. Using a random-effects model, they assessed outcomes such as all-cause mortality, reinfarction, repeat revascularization, and MACE over a mean follow-up of approximately 31 months. In the short term, complete and culprit-only PCI showed no significant differences in mortality or periprocedural myocardial infarction, indicating comparable early safety. However, during longer-term follow-up, clear prognostic benefits of CR emerged: it was associated with significantly lower all-cause mortality (RR 0.66, 95% CI 0.55–0.79), reinfarction (RR 0.57, 95% CI 0.43–0.76), repeat revascularization (RR 0.60, 95% CI 0.54–0.66), and a reduced risk of MACE (RR 0.72, 95% CI 0.63–0.82).

When these individual studies are considered together, the apparent discrepancies become more understandable: early procedural risks may offset short-term benefits, whereas the reduction in residual ischemic burden becomes more evident during longer follow-up.

Taken together, the studies included delineate a consistent message: in patients with NSTEMI and multivessel coronary artery disease, complete revascularization generally provides a clear clinical advantage over a culprit-only approach, with benefits that become increasingly evident over long-term follow-up. Large multicenter registries such as BleeMACS, extensive observational analyses including those by Rathod and Pustjens, as well as single-center cohorts from Portugal and Asia converge on the finding that treating all significant lesions reduces recurrent ischemia, reinfarction, and the need for repeat revascularization, and in many datasets is also associated with improved long-term survival. Although some studies—such as the cohort by Li and those involving earlier-generation stents—did not demonstrate a mortality advantage, they nonetheless confirm that complete revascularization significantly lowers the burden of residual ischemic disease and the likelihood of future interventions. Additionally, the timing data suggest that the early procedural risk associated with more extensive intervention is transient, whereas the long-term benefits of complete treatment consistently emerge after the initial months of follow-up. Overall, the collective evidence supports the conclusion that, in NSTEMI patients with multivessel disease, complete revascularization offers the most favorable prognostic profile when anatomically and clinically feasible. Despite variability in patient risk, lesion complexity, and procedural practice across studies, the overarching message is coherent: decisions regarding the extent of revascularization meaningfully influence long-term outcomes, and complete revascularization should be the preferred goal whenever circumstances allow.

Table 1 presents the principal baseline and procedural characteristics across included studies investigating complete versus incomplete revascularization in NSTEMI and multivessel coronary disease.

Having established the clinical relevance of complete versus incomplete revascularization, the discussion next addresses the timing of this strategy, since the benefit of treating non-culprit lesions may depend on whether PCI is performed immediately, during the same hospitalization, or at a later staged procedure.

Equally important is the question of timing, as growing evidence suggests that the prognostic value of revascularization may differ substantially depending on whether it is performed immediately during the index procedure or deferred as a staged intervention.

The only prospective, randomized, multicenter controlled trial conducted exclusively in patients with NSTEMI, the SMILE trial by Sardella et al. [60], which included 584 patients with NSTEMI and MVD, evaluated the advantage of complete revascularization in single-stage vs. multistage PCI in NSTEMI with MVD. Single stage PCI treated all lesions during the index procedure, whereas multi-stage PCI complete revascularization in subsequent sessions during the same hospitalization (second procedure between 3–7 days from index procedure). Inclusion criteria were age >18 years, glomerular filtration rate >60 mL/min, planned early invasive strategy. Patients with cardiogenic shock, significant unprotected left main coronary artery disease (typically > 50% stenosis), previous or candidate for CABG, CTO, severe comorbidities were excluded from the study. Baseline clinical characteristics were well balanced between groups, as well as angiographic features, with an average of ~2.3 vessels treated, a SYNTAX score of 15–16, and similar lesion complexity (type C lesions 36–38%). Drug-eluting stents were used in >75% of cases, with no difference in stent type, number (median 3), or cumulative stent length (24 mm). Primary end-point was MACCE—a composite of cardiac death, all cause death, reinfarction, rehospitalization for UA, repeat coronary revascularization and stroke for up to one year. At 1 month, event rates were low in both arms: MACCE occurred in 3.0% of the single-stage group versus 2.3% of the multistage group, with no significant differences in death, myocardial infarction, or target vessel revascularization. The only early signal was a higher incidence of minimal bleeding in the multistage arm (2.3% vs. 0%, *p* = 0.03). By 6 months, differences became more evident, with MACCE significantly higher in the multistage group (10.6% vs. 5.7%, *p* = 0.04), a strong trend toward increased all-cause mortality (7.6% vs. 3.8%, *p* = 0.06), and numerically more cardiac deaths and revascularizations. At one year, one-stage PCI was superior to multistage PCI for reducing MACCE at one year (one-stage PCI: *n* = 36 [13.63%] vs. multistage-PCI: *n* = 61 [23.19%]; hazard ratio [HR]: 0.549 [95%CI: 0.363 to 0.828]; *p* = 0.004) and for reducing the incidence of target vessel revascularization at one year (one-stage PCI: *n* = 22 [8.33%] vs. multi-stage PCI: *n* = 40 [15.20%]; HR: 0.522 [95% CI: 0.310 to 0.878]; *p* = 0.01), along with lower minimal bleeding rates, a more rapidly dropping of troponin T levels among patients with single-stage PCI (while as multi-stage PCI patients had a delayed decrease and even an increase in troponin after the staged ischemia, indicating prolonged ischemia). Mortality showed a trend favoring single-stage PCI (6.4% vs. 11.0%, *p* = 0.06), though cardiac death (3.4% vs. 5.3%) and myocardial infarction (2.7% vs. 3.8%) were not statistically different. Despite initial concerns, one-stage PCI was not associated with higher complication rates such as periprocedural MI, stroke or contrast induced nephropathy. Overall, the SMILE trial demonstrates that complete revascularization is beneficial in NSTEMI with multivessel disease and that performing it during a single index procedure, rather than staging it, is associated with superior clinical outcomes. The evidence clearly favors one-stage complete revascularization as the optimal strategy.

A prespecified analysis of the BIOVASC trial (Biomarker-guided Immediate Versus Staged Multivessel Revascularization in Acute Coronary Syndromes) [61,62] investigated the optimal timing of complete revascularization in patients presenting with NSTE-ACS and MVD. The primary objective of this sub-study was to compare immediate complete revascularization (ICR), performed during the index coronary procedure, with staged complete revascularization (SCR), in which treatment of non-culprit lesions was deferred and completed within six weeks of the index PCI. Out of the 1525 patients enrolled in the main BIOVASC trial, 917 had NSTE-ACS. These patients were randomized to either ICR (*n* = 459) or SCR (*n* = 458). The primary outcome was the composite of all-cause mortality, myocardial infarction, any unplanned ischemia-driven revascularization, or cerebrovascular events at 1 year after the index procedure. At 30 days, ICR was associated with a significantly lower incidence of the primary composite endpoint compared with SCR (1.8% vs. 5.7%; risk difference [RD] 4.0%; 95% CI 1.5–6.4; *p* = 0.001). Similarly, the composite of cardiovascular death or myocardial infarction occurred in 0.4% of ICR versus 3.3% of SCR patients (RD 2.9%; 95% CI 1.1–4.6; *p* = 0.001), while the incidence of myocardial infarction alone was 0.2% versus 3.1%, respectively (RD 2.9%; 95% CI 1.2–4.5; *p* < 0.001). Unplanned ischemia-driven revascularization was also reduced in the ICR group (0.9% vs. 3.7%; RD 2.9%; 95% CI 0.9–4.8; *p* = 0.004), as was the broader composite of death, MI, stroke, or major bleeding (1.3% vs. 5.7%; RD 4.4%; 95% CI 2.0–6.8; *p* < 0.001). Importantly, all type 1 MIs between index and staged procedures occurred in patients with NSTE-ACS allocated to SCR. At one year however, the primary composite endpoint did not differ significantly between groups (7.9% vs. 10.1%; RD 2.2%; 95% CI −1.5 to 6.0; *p* = 0.15). Cardiovascular mortality was comparable (1.1% vs. 0.9%; RD −0.2%; 95% CI −1.5 to 1.1; *p* = 0.75), but ICR remained superior in reducing myocardial infarction (2.0% vs. 5.3%; RD 3.3%; 95% CI 0.9–5.7; *p* = 0.006) and unplanned revascularization (4.2% vs. 7.8%; RD 3.5%; 95% CI 0.4–6.6; *p* = 0.018). Piecewise Cox regression analyses confirmed that the advantage of ICR was concentrated in the early post-PCI phase: within 30 days, ICR significantly reduced the risk of the primary outcome (HR 0.29; 95% CI 0.13–0.65; *p* = 0.003), myocardial infarction (HR 0.07; 95% CI 0.02–0.52; *p* = 0.010), and unplanned revascularization (HR 0.23; 95% CI 0.08–0.68; *p* = 0.008), while no differences were observed between 31 and 365 days. These results indicate that the clinical advantage of ICR is largely an early benefit, particularly in NSTE-ACS patients, in whom delaying non-culprit revascularization may expose them to preventable ischemic events.

The recent 2-year BIOVASC analysis demonstrated no sustained benefit of ICR over a staged approach in either STE- or NSTE-ACS patients, with treatment effects converging after the first year [63]. While the 1-year NSTEMI sub-study suggested that ICR reduced non-procedural MI and unplanned revascularization, the extended follow-up revealed a late catch-up phenomenon in the NSTEMI cohort, largely driven by higher rates of unplanned revascularization. The authors highlight that ICR was performed with significantly less use of intracoronary imaging, particularly in NSTE-ACS, which may have led to suboptimal lesion assessment, stent deployment, or missed high-risk plaques, thereby reducing the long-term durability of ICR. Both strategies were safe, with no increase in bleeding, stroke, or mortality with ICR. Differences compared with the MULTISTARS-AMI trial [64], which showed clearer benefit in STEMI—a significant reduction in risk of myocardial infarction and unplanned ischemia driven revascularization when patients were randomized to ICR-, may relate to variations in endpoint definitions and trial design. Taken together, the findings suggest that ICR is safe and can reduce very early ischemic events in NSTEMI, but its durability beyond 1 year appears less certain.

These randomized data therefore introduce an important temporal dimension: immediate complete revascularization may protect against early ischemic events, but its long-term durability appears to depend on careful lesion assessment and procedural optimization.

An overview of randomized controlled trials examining the timing of PCI in NSTEMI/ACS with multivessel disease is provided in Table 2.

Beyond large, randomized trials and registries, several observational studies from different regions have provided complementary real-world insights into PCI revascularization timing in NSTEMI with multivessel disease.

This real-world evidence is particularly useful because it reflects the complexity of daily NSTEMI practice, where timing decisions are influenced by hemodynamic stability, renal function, lesion complexity, and operator judgment.

Rao et al. [65] conducted a multicenter cohort study including 2460 consecutive NSTEMI patients with MVD across five institutions in China. They compared outcomes between CR versus IR, with a focus on both timing and strategy. The primary endpoints were MACCEs and MACEs. Over a median follow-up of approximately 528 days, complete revascularization was associated with a substantially lower risk of both MACCEs (adjusted hazard ratio [aHR] 0.48, 95% CI 0.36–0.64) and MACEs (aHR 0.45, 95% CI 0.33–0.60) compared to incomplete revascularization. When comparing single-stage versus multistage complete revascularization, outcomes were similar (MACCEs: aHR 0.93, MACEs: aHR 0.89). However, early multistage revascularization conferred a significantly greater benefit than delayed multistage revascularization—dramatically reducing MACCEs (aHR 0.05, 95% CI 0.01–0.28) and MACEs (aHR 0.03, 95% CI 0.01–0.27). This multicenter observational study demonstrates that complete revascularization in NSTEMI with multivessel disease significantly lowers adverse clinical events compared to incomplete revascularization. Importantly, early staged revascularization appears to be superior to delayed approaches, suggesting that both the extent and timing of intervention critically influence long-term outcomes.

In a single-center retrospective cohort of 298 NSTEMI patients with MVD undergoing complete revascularization, Alici et al. [66] compared single-stage PCI (SS-PCI, *n* = 71; 23.8%) versus multi-stage PCI (MS-PCI, *n* = 227; 76.2%), assessing short-term (in-hospital) and long-term (~24-month) mortality. The SS-PCI group presented with more complex anatomy and higher SYNTAX score, *p* = 0.001, longer cumulative stent length 32 vs. 28 mm, *p* = 0.037, more left-main culprit lesions (7.0% vs. 0.4%) and more LAD culprits (53.5% vs. 43.2%), while RCA culprits were less frequent (15.5% vs. 30.0%) (*p* = 0.001 for distribution). They also arrived with worse baseline flow (TIMI 0: 29.6% vs. 4.4%; TIMI 3: 40.8% vs. 63.9%; *p* < 0.001) and, despite good final angiographic results, had fewer final TIMI 3 flows (84.5% vs. 94.7%; *p* = 0.016) and more no-reflow (15.5% vs. 5.3%; *p* = 0.005); adjunctive therapy was used more intensively with SS-PCI (pre-dilatation 77.5% vs. 50.2%, *p* < 0.001; tirofiban 14.1% vs. 4.0%, *p* = 0.005; DES 80.3% vs. 62.6%, *p* = 0.006). On outcomes, in-hospital mortality was higher univariately with SS-PCI (14.1% vs. 4.0%; *p* = 0.005), but PCI strategy was not an independent predictor of total (long-term) mortality after multivariable modeling; instead, lower hemoglobin predicted death (OR 0.485 per g/dL; *p* = 0.002), no-reflow carried a strong adverse signal (OR 6.19; *p* = 0.021), and omitting post-dilatation was associated with harm (OR 0.287; *p* = 0.045; protective when performed). The authors concluded that, when complete revascularization is pursued in NSTEMI-MVD, anatomy and procedural quality (avoiding no-reflow, optimizing post-dilatation) rather than staging per se drive prognosis.

Also, in a post hoc analysis of FRAME-AMI [67], investigators studied 549 AMI patients with multivessel disease who all underwent CR during the index hospitalization, comparing ICR (*n* = 329; 60%) with SCR (*n* = 220; 40%) over a median 3.48 years of follow-up. Patients selected for ICR had modestly different baselines (fewer men (81.2% vs. 89.1%), higher SBP (130 vs. 125 mmHg), more diabetes (36.5% vs. 26.4%), lower creatinine (0.9 vs. 1.0 mg/dL), and slightly higher LVEF (54% vs. 53%)) and, angiographically, showed less three-vessel disease (31.6% vs. 48.6%) with a different culprit distribution (LAD 40.4% vs. 26.4%; RCA 33.4% vs. 54.5%). Procedurally, ICR used fewer total stents (2.3 vs. 2.5) and shorter non-culprit stent length (34.8 vs. 43.8 mm), relied less on a transfemoral approach (9.7% vs. 25.5%), and used imaging for non-culprit PCI less often (24.9% vs. 36.8%). In-hospital complications were similarly low (3.6% vs. 6.4%) aside from less cardiogenic shock in the immediate group (0.3% vs. 2.7%). The primary endpoint (composite of all-cause death, MI, or any repeat revascularization) did not differ between strategies in the overall cohort (12.7% vs. 17.4%; adjusted HR for staged vs. immediate 0.81, 95% CI 0.43–1.53; *p* = 0.528), and no secondary endpoint (each component, stroke, stent thrombosis, contrast induced nephropathy) showed a significant difference. Results were consistent in STEMI and NSTEMI subgroups. When stratified by presentation, ICR was chosen far more often in NSTEMI patients (75%) compared with STEMI patients (42%). Among STEMI cases, those treated immediately were more likely to have diabetes (35% vs. 21%), had more LAD culprit lesions and fewer RCA culprits, and required shorter stent lengths and fewer stents for non-culprit arteries. In NSTEMI, similar patterns were observed, with ICR patients more likely to have LAD involvement (37% vs. 20%), less RCA involvement (35% vs. 50%), and shorter stents used (35 vs. 42 mm). Interestingly, door-to-balloon times were longer in NSTEMI patients undergoing immediate PCI (over 10 h vs. around 6 h), likely reflecting timing of decision-making. Collectively, within an index-admission CR strategy, timing (immediate vs. staged) did not influence clinical outcomes, while case-mix and lesion complexity drove procedural differences, suggesting that either timing is reasonable when CR is pursued, provided anatomy and patient factors are suitable.

In a retrospective, single-center study from Tianjin Chest Hospital [68] were evaluated the long-term outcomes of three PCI strategies in 943 hemodynamically stable patients with NSTEMI and MVD treated with new-generation DES. Patients were categorized into CO-PCI, immediate MV-PCI during the index procedure and out-of-hospital staged MV-PCI, performed within 60 days after discharge. The primary endpoint was MACE: all-cause death, MI, repeat revascularization. Median follow-up was 59 months. Immediate MV-PCI reduced all-cause mortality compared with CO-PCI (HR 0.59, *p* = 0.034). Staged MV-PCI was associated with lower rates of MACE (HR 0.45, *p* < 0.001) and all-cause death (HR 0.33, *p* < 0.001) compared with CO-PCI. When directly compared, staged MV-PCI lowered the risk of MACE and repeat revascularization compared to immediate MV-PCI, particularly in patients with complex coronary disease (SYNTAX score > 22). Independent predictors of worse outcomes included age >65, diabetes, low LVEF, and renal dysfunction, while multivessel PCI was independently associated with reduced mortality. In this cohort, immediate MV-PCI was more often chosen for patients with higher ischemic risk (higher GRACE, more left main disease), CO- PCI for those with renal dysfunction, and staged MV-PCI for patients with more complex anatomy (type B2/C lesions, longer stents), underscoring that strategy selection reflected both clinical risk and lesion complexity. These distinctions are highly relevant for the ongoing debate on optimal PCI strategy in NSTEMI with MVD. They illustrate that procedural choice is not random but influenced by patient risk profile, lesion complexity, and operator judgment. Importantly, the fact that staged PCI was preferentially used in anatomically complex cases, yet still associated with better outcomes, strengthens the argument that timing and individualized strategy may be as critical as completeness of revascularization. MV-PCI, whether immediate or staged, offered survival benefits over culprit-only intervention in NSTEMI patients with MVD. Importantly, staged revascularization after discharge provided the most favorable long-term outcomes, especially in anatomically complex cases, highlighting the potential role of tailored timing in PCI strategy. As limitations, the study’s retrospective, single-center design introduces selection bias; the sample size was relatively small with 15% loss to follow-up; intravascular imaging and physiology-guided assessment were rarely used; and data on procedure duration, contrast-induced nephropathy, radiation exposure were not collected.

Demirkiran et al. [69] conducted a retrospective, single-center cohort study that evaluated the long-term outcomes (up to 10 years) of different revascularization strategies in patients with NSTEMI and MVD. From a screened population of over 14,000 ACS patients (2014–2024), a total of 316 NSTEMI patients with at least one angiographically significant non-infarct related artery (non-IRA) lesion were included. Patients with STEMI, unstable angina, prior revascularization, advanced renal dysfunction, or hemodynamic instability were excluded to reduce confounding. Patients were stratified into three groups: ICR—all significant lesions treated during the index PCI; SCR—complete revascularization subdivided into ≤24 h vs. >24 h between index and staged procedure and non-complete revascularization (NCR)—only the IRA treated, either due to patient refusal or clinical judgment. As procedural characteristics PCI was performed with drug-eluting stents; procedural success was defined as <30% residual stenosis; the choice of stent length, diameter, vascular access, and use of glycoprotein IIb/IIIa inhibitors was left to operator discretion; stent length tended to be longer in the ICR group compared to SCR/NCR; on average, patients in the complete revascularization groups received three stents (IQR 2–5), while the NCR group received only one or two; all patients received DAPT for at least one year, followed by aspirin monotherapy; lesion significance was assessed angiographically. Over a median follow-up of 63 months (up to 10 years), ICR demonstrated the most favorable long-term outcomes, with significantly lower rates of cardiac composite events (12.8% vs. 36.5% SCR vs. 40.7% NCR, *p* = 0.001), recurrent MI (9.6% vs. 36.5% vs. 39.8%, *p* = 0.001), cardiac death (3.8% vs. 13.5% vs. 15.7%, *p* = 0.003), all-cause mortality (7.1% vs. 26.9% vs. 23.1%, *p* = 0.002), and rehospitalization (16.7% vs. 50% vs. 41.7%, *p* < 0.001). Importantly, no significant differences were observed between SCR and NCR overall (HR 1.06, 95% CI 0.61–1.84; *p* = 0.832). However, timing within the SCR cohort was crucial: patients undergoing staged PCI within 24 h had outcomes comparable to ICR (cardiac events 16.7%), while those with a delay beyond 24 h fared significantly worse (47.1%, *p* = 0.038). Landmark analysis revealed that the survival benefit of ICR became especially evident after 20 months of follow-up. These findings suggest that both the completeness and timeliness of PCI are critical in NSTEMI with MVD. The favorable results of ICR and early SCR may be explained by reducing the risk of misidentifying the culprit lesion in NSTEMI, by treating vulnerable non-culprit plaques that may rupture and cause recurrent events and by minimizing the “at-risk window” between index and staged interventions.

Overall, these observational studies suggest that timing should be viewed as a flexible component of an individualized revascularization plan rather than as a fixed rule. Immediate or early staged PCI may be appropriate when clinical and anatomical conditions are favorable, whereas delayed intervention may be reserved for selected complex cases.

An overview of the observational evidence on timing strategies for PCI in NSTEMI with multivessel disease is presented in Table 3.

Following the randomized and observational evidence, the next step is to consider how pooled analyses integrate these heterogeneous findings and what limitations remain when STEMI and NSTEMI populations are combined.

Systematic reviews and meta-analyses have reinforced the advantage of complete revascularization in patients with multivessel coronary artery disease, but it is important to note that these syntheses predominantly draw on trials including both STEMI and NSTEMI populations. As a result, the signal of benefit is clear at the global level of acute myocardial infarction yet remains less well defined for NSTEMI patients specifically.

Reddy et al. [70] conducted a large-scale systematic review and network meta-analysis of 24 RCTs including 16,371 patients with acute MI (both STEMI and NSTEMI) and multivessel disease. The weighted mean follow-up was 26 months. The populations were generally younger (mean age in most trials <65 years), predominantly male (>75%), and with lower prevalence of diabetes and chronic kidney disease (CKD) compared with real-world NSTEMI cohorts. Their analysis demonstrated that complete revascularization was superior to culprit-only PCI, with significant reductions in all-cause mortality (RR 0.85), cardiovascular death, recurrent MI, MACE, and repeat revascularization, without increased risk of bleeding, acute kidney injury, or stent thrombosis. Immediate complete revascularization ranked higher than staged approaches. However, because most included trials enrolled STEMI patients, extrapolation to NSTEMI remains limited.

Bianchini et al. [71] conducted a comprehensive systematic review and meta-analysis comprising 22 observational studies and 182,798 patients with NSTE-ACS and MVD. The analysis compared CR—performed during the index procedure or hospitalization—versus CO revascularization. In the propensity-matched population (11,372 patients; mean age 68.7 years, 59% men, 27% with diabetes, 5.1% with CKD and about one-third with three-vessel disease), CR was associated with a 28% reduction in all-cause mortality (OR 0.72, 95% CI 0.58–0.90), a 35% reduction in major adverse cardiovascular events (MACE) (OR 0.65, 95% CI 0.47–0.88), and a 60% reduction in recurrent myocardial infarction (OR 0.40, 95% CI 0.25–0.63) compared with CO revascularization. Short-term analyses showed no significant differences in mortality or MACE, although a slight increase in periprocedural myocardial infarction was observed among patients undergoing CR, likely reflecting procedural complexity and more extensive lesion treatment. CR also conferred lower rates of repeat revascularization (41%) and re-PCI (33%), particularly in those with non-culprit lesions. Meta-regression revealed that the benefits of CR were most pronounced in patients with three-vessel disease, while the advantage was less marked among diabetics. Despite moderate heterogeneity and the observational nature of most included studies, the results were consistent across sensitivity analyses. Overall, this meta-analysis supports the concept that CR is associated with improved long-term clinical outcomes in NSTE-ACS patients with MVD, particularly in those with extensive coronary involvement, preserved renal function, and without hemodynamic instability.

Chen et al. [72] performed the first network meta-analysis restricted to NSTEMI with MVD, analyzing 8 observational studies involving 34,151 patients. Unlike the other reviews, Chen compared four strategies: CO-PCI, ICR, SCR, and planned MV-PCI during a second hospitalization. They reported that MV-PCI was associated with the lowest all-cause mortality (OR 0.53), cardiovascular mortality (OR 0.48), MACE (OR 0.53), and repeat revascularization (OR 0.55). In contrast, ICR showed the greatest reduction in recurrent MI (OR 1.39 vs. COR). CO-PCI consistently performed worst across outcomes. This nuanced comparison highlighted that both ICR and MV-PCI are superior to culprit-only approaches, but the balance between reducing recurrent MI (favoring ICR) and lowering long-term mortality and revascularization (favoring MV-PCI) remains unresolved.

This network-level perspective helps reconcile the evidence: complete revascularization appears consistently preferable to culprit-only PCI, but the optimal timing of completion remains dependent on the clinical endpoint, patient profile, and lesion characteristics considered.

Overall, the totality of evidence indicates that complete revascularization in NSTEMI with multivessel disease is both feasible and beneficial, with signals toward reduced myocardial infarction, unplanned revascularization, and long-term mortality compared with CO-PCI. However, robust, adequately powered, multicenter randomized trials dedicated exclusively to NSTEMI patients are still warranted to confirm these benefits, to better define which subgroups derive the greatest net advantage, and to establish standardized approaches for timing, lesion assessment, and procedural strategy.

The ongoing COMPLETE-NSTEMI trial [73] is a landmark prospective, randomized, multicenter study designed to determine whether complete multivessel PCI offers superior outcomes compared with culprit-lesion-only PCI in patients presenting with NSTEMI and concomitant MVD. Enrolling approximately 3390 patients across 65–70 centers in Germany and Austria, participants are randomized in a 1:1 fashion to either complete revascularization or a culprit-only strategy. The primary endpoint is a composite of cardiovascular death or rehospitalization due to nonfatal myocardial infarction, and the trial is event-driven, targeting 578 primary events with a minimum of 12 months follow-up per patient. Recruitment began in October 2023, with over 500 patients randomized by April 2025, and completion anticipated in the first half of 2027, while results are expected in 2028.

Consequently, the current evidence should be interpreted as supporting a structured but individualized approach rather than a universal timing algorithm.

Overall, the accumulated evidence indicates that timing meaningfully influences outcomes in NSTEMI patients with multivessel disease, complementing the benefit of complete revascularization. The SMILE trial clearly demonstrated the superiority of single-stage complete PCI, while the BIOVASC NSTE-ACS substudy showed that immediate revascularization provides substantial early protection—particularly against myocardial infarction and unplanned revascularization—although its advantage diminishes beyond the first year. Observational studies further suggest that both immediate and early staged (≤24–72 h) approaches are safe and effective, whereas delayed staging consistently yields worse outcomes. Meta-analyses by Reddy, Bianchini, and Chen reinforce that complete revascularization improves long-term prognosis, with immediate strategies offering the greatest early ischemic benefit and staged approaches in selected patients contributing to durable long-term results. In summary, while no single timing strategy is universally optimal, the literature supports aiming for complete revascularization performed either immediately or early, tailored to clinical stability, lesion complexity, and procedural considerations. Pending results from COMPLETE-NSTEMI, individualized timing—rather than rigid protocols—remains the most evidence-aligned approach for NSTEMI with MVD.

#### 4.2.3. The Role of Age in Guiding PCI Strategy in NSTEMI and MVD

Revascularization strategies among patients with NSTE and MVD depend also on the frailty status of the patients, considering that most patients are older and with multiple comorbidities, as compared to STEMI patients.

The FIRE trial [74], a multicenter randomized study, investigated the optimal revascularization strategy in patients aged 75 years or older presenting with acute myocardial infarction (both STEMI and NSTEMI) and multivessel coronary artery disease. A total of 1445 patients were randomized to receive either CO- PCI or physiology-guided CR with non-culprit lesions evaluated by fractional flow reserve or angiographic severity. Over a median follow up of one year, a composite primary endpoint (all cause death, MI, stroke or ischemia driven revascularization) occurred significantly less often in the CR group (15.7%) compared to CO group (21.0%). Notably, the benefit was observed consistently in the NSTEMI subgroup, which represented approximately 65% of study population. The secondary composite outcome of cardiovascular death or MI was also significantly reduced. Importantly, the incidence of safety outcomes such as stroke, bleeding and contrast induced nephropathy did not differ significantly between groups. These findings support a strategy of physiology-guided complete revascularization in elderly patients with NSTEMI and multivessel disease, challenging due to prior tendency toward minimalist or culprit-only PCI in this age group. The study emphasizes that age alone should not preclude a comprehensive PCI strategy when clinically appropriate. Recently, a 3-year follow-up analysis of FIRE trial [75] confirmed that these benefits are durable, with sustained reductions in major adverse cardiovascular events, cardiovascular death, and recurrent MI. Alongside, fewer hospitalizations for heart failure, without excess bleeding or procedural complications were noticed. For NSTEMI patients these findings underscore that complete, physiology-guided revascularization not only improves early outcomes but also provides lasting clinical protection, thereby reinforcing its role as a preferred strategy in this subgroup.

A multicenter observational study [76] pooled registry data from four cohorts to evaluate revascularization strategies in patients aged ≥75 years presenting with AMI (STEMI or NSTEMI) and MVD, who had undergone successful culprit-lesion treatment. A total of 2087 patients were included; 65% received a CO-PCI, while 35% underwent CR. Propensity scores were calculated using logistic regression with backward selection (*p* < 0.2), and analyses incorporated inverse probability of treatment weighting and multivariable adjustment to minimize confounding. The primary endpoint was all-cause mortality at approximately one year (mean follow-up 419 days). Secondary endpoints included cardiovascular death, recurrent MI, and major bleeding. Mean patient age was 81.5 years. After risk adjustment, CR was associated with significantly lower 1-year all-cause mortality (adjusted rate 9.7% vs. 12.9%; HR 0.67; 95% CI 0.50–0.89). Similarly, rates of cardiovascular death (HR 0.68; 95% CI 0.48–0.95) and re-infarction (HR 0.67; 95% CI 0.48–0.95) were significantly lower in the complete strategy group. Major bleeding rates did not differ significantly between groups. The findings from this observational analysis provide compelling evidence that CR may be associated with improved clinical outcomes in older patients (≥75 years) presenting with myocardial infarction and multivessel coronary artery disease. The observed reductions in all-cause mortality, cardiovascular death, and recurrent myocardial infarction in the CR group suggest a potential benefit of addressing the full ischemic burden rather than limiting intervention to the culprit lesion alone. Importantly, this benefit did not come at the cost of increased major bleeding, which is particularly relevant in elderly populations who often carry elevated procedural risk. However, these results must be interpreted in the context of the study’s limitations. The observational design introduces the possibility of selection bias, particularly as patients selected for complete revascularization may have been healthier or more functionally robust (factors not fully captured in the dataset). The absence of frailty assessments and other geriatric-specific variables, such as cognitive status, functional dependence, or patient preferences, further limits the generalizability of the findings to a frailer population. Additionally, the timing and completeness of revascularization were not standardized, which introduces procedural heterogeneity. Despite these limitations, the consistent benefit observed across multiple ischemic outcomes supports the consideration of complete revascularization in older adults with multivessel disease, provided that clinical judgment, frailty status, and individualized risk–benefit assessments are carefully incorporated into the decision-making process.

In a multicenter retrospective cohort study, Rocha de Almeida et al. [77], investigated the outcomes of CR versus CO revascularization in elderly patients aged 75 years and older presenting with acute coronary syndrome and multivessel coronary artery disease. The cohort included 629 consecutive patients treated between 2015 and 2019 across five tertiary centers in Brazil. Eligible participants were ≥75 years old and had angiographically confirmed multivessel coronary artery disease, with at least one significant non-culprit lesion in addition to the culprit vessel. Patients were stratified retrospectively based on the in-hospital revascularization approach: 129 patients (34%) received CR (defined as treatment of all significant lesions during the index hospitalization), while 254 patients (66%) were managed with CO-PCI. The primary outcomes of interest were in-hospital mortality and MACE, with long-term follow-up extending to a median of 1073 days to assess all-cause mortality, cardiovascular hospitalizations, and a composite of both. The findings revealed a statistically significant reduction in adverse in-hospital outcomes among patients undergoing CR. In-hospital mortality was markedly lower in the CR group compared to the CO group (6% vs. 19%, respectively; odds ratio [OR] 0.30, 95% CI 0.13–0.71), and CR was also associated with a lower rate of MACE (34% vs. 51%, OR 0.62, 95% CI 0.40–0.96). However, during long-term follow-up, no significant differences emerged between the two groups in terms of all-cause mortality, cardiovascular readmissions, or the composite outcome of death and cardiovascular hospitalization. These neutral long-term results suggest that while CR may reduce early in-hospital events, the benefit does not clearly extend into the chronic phase post-discharge. The authors discussed several potential explanations for this divergence between short- and long-term outcomes. One hypothesis is that while CR may stabilize patients acutely by eliminating multiple ischemic foci, the progression of underlying atherosclerotic disease or competing risks associated with age and frailty may diminish the relative benefit of CR over time. Additionally, the lack of standardized use of physiological lesion assessment (e.g., fractional flow reserve) or intracoronary imaging to guide PCI may have led to overtreatment or undertreatment of non-culprit lesions in the CR group. Moreover, since the decision for CR versus CO was left to the discretion of the treating physician, selection bias may have favored more robust or hemodynamically stable patients for CR, further complicating interpretation. Importantly, although the study was not restricted to NSTEMI patients, the majority of included individuals had a non-ST-elevation presentation, rendering the results particularly relevant for clinicians managing NSTEMI with multivessel disease in the elderly. When considered alongside emerging randomized evidence such as the FIRE trial (which demonstrated that physiology-guided CR significantly reduced cardiovascular mortality and ischemic events in elderly ACS patients), the results of this observational study provide complementary support for a more proactive approach to MV-PCI in selected older individuals.

Agra-Bermejo and colleagues [78] assessed the long-term impact of CR versus CO-PCI in 1722 elderly patients (≥75 years) with NSTEMI and multivessel disease admitted across two Spanish centers, between 2003 and 2016. Only 30.4% received CR, while 69.6% underwent culprit-only intervention. Patients in the CR group were slightly younger (80.7 vs. 81.7 years), more often male (36.8% vs. 41.7% female), and had fewer comorbidities: lower prevalence of prior CAD (28.8% vs. 36.9%), previous MI (10.5% vs. 19.4%), prior CABG (2.7% vs. 9.3%), and heart failure (6.1% vs. 11.0%). They also had lower GRACE (154 vs. 163) and CRUSADE scores (27 vs. 32) compared with culprit-only patients, suggesting a slightly lower baseline risk profile. After propensity-score matching (500 vs. 500 patients), groups were well balanced. At a median follow-up of 45.7 months, CR was associated with significantly reduced all-cause mortality (30.5% vs. 44.5%, HR 0.74, 95% CI 0.57–0.97) and cardiovascular mortality (17.4% vs. 32.6%, HR 0.67, 95% CI 0.47–0.94) compared with CO-PCI. In-hospital mortality was also lower with CR (2.5% vs. 7.3%). However, no significant differences emerged in terms of MACE (56.1% overall, *p* = 0.28) or recurrent ACS (*p* = 0.58). Multivariate analysis confirmed CR as an independent protective factor for mortality, while older age, prior CAD, diabetes, heart failure, and higher GRACE score predicted worse outcomes.

A retrospective registry-based study [79] examined the impact of CR versus IR in 1018 elderly patients (≥75 years) with ACS and MVD undergoing PCI between 2011 and 2019 in Taiwan. Of these, 496 (48.7%) received CR and 522 (51.3%) underwent IR. Patients in the IR group were slightly older (80.1 vs. 79.4 years), had lower LVEF (55.0% vs. 58.5%), and more frequently presented with STEMI (31.4% vs. 24.0%). Other baseline factors (sex, diabetes, hypertension, CKD, prior MI, frailty) were similar after propensity-score matching (395 pairs), yielding well-balanced groups. At 3-year follow-up, CR was associated with significantly lower MACE rates (16.7% vs. 25.6%, HR 0.65, 95% CI 0.47–0.88, *p* = 0.006), driven mainly by reduced all-cause mortality (7.6% vs. 18.0%, HR 0.38, 95% CI 0.24–0.62, *p* < 0.001). No significant differences were observed for recurrent non-fatal MI (2.3% vs. 3.0%) or repeat revascularization (10.9% vs. 11.9%). Subgroup analyses confirmed consistent benefit across age, diabetes, CKD, and frailty strata, with a particularly strong effect in STEMI patients, though in NSTE-ACS patients, CR lowered all-cause mortality (HR 0.39) and cardiac mortality (HR 0.30) but did not significantly reduce overall MACE.

In older patients with NSTEMI and multivessel disease, revascularization strategy cannot be separated from frailty and biological age, yet the emerging data consistently challenge a reflex “culprit-only” approach in this population. The FIRE trial showed that in patients ≥75 years, most of whom presented with NSTEMI, physiology-guided complete revascularization significantly reduced death, MI, stroke, and ischemia-driven revascularization versus culprit-only PCI, with sustained benefit at 3 years and no excess in bleeding or procedural complications. Large multicenter registries and cohorts in elderly AMI/NSTEMI patients largely echo these findings: complete revascularization is repeatedly associated with lower all-cause and cardiovascular mortality and less reinfarction, without a clear penalty in major bleeding, even though some studies suggest that the advantage may be most pronounced in the early and mid-term rather than uniformly durable over very long follow-up. However, these observational data are unavoidably influenced by selection bias, with more robust, less comorbid, and less frail patients more likely to be offered complete revascularization. Formal frailty indices, cognition, functional dependence, and patient preferences were rarely captured, limiting generalizability to the frailest phenotypes. Taken together, the available evidence supports that, in elderly NSTEMI patients with multivessel disease, age alone should not preclude complete revascularization, which appears both feasible and beneficial in carefully selected individuals. At the same time, decisions must remain highly individualized, explicitly integrating frailty status, life expectancy, comorbidity burden, and goals of care into the choice between complete versus culprit-only PCI.

## 5. Conclusions

The management of NSTEMI in patients with multivessel coronary artery disease remains a subject of ongoing debate. Unlike STEMI, where randomized evidence has clearly demonstrated the benefits of complete revascularization, the data in NSTEMI are less consistent and are largely derived from registries, observational studies, and a limited number of randomized trials. This variability reflects the greater heterogeneity of NSTEMI populations, who are typically older, more comorbid, and often present with more diffuse and complex coronary disease.

Available evidence increasingly supports complete revascularization as a strategy that reduces long-term mortality, recurrent ischemia, and the need for subsequent procedures. However, these benefits must be interpreted in the context of higher periprocedural risks, longer procedures, and greater contrast exposure. Patient characteristics, such as age, frailty, renal function, and diabetes, further influence the net benefit of a complete strategy. These high-risk subgroups are simultaneously the most likely to benefit from ischemic protection and the most vulnerable to complications, underscoring the importance of individualized treatment planning. Advances in PCI technology and adjunctive pharmacotherapy have improved the safety profile of more extensive interventions, making complete revascularization more feasible in contemporary practice.

The relatively limited number of studies included, specifically evaluating revascularization strategies in NSTEMI patients with multivessel coronary disease, should be acknowledged as an important limitation. This restricted evidence base may reduce the external validity of the findings and limits the ability to formulate broadly generalizable recommendations. However, this limitation also underscores the clinical relevance of the present review, as it highlights an important area in which evidence remains less robust than in STEMI. Despite differences in study design, patient selection, revascularization definitions, timing strategies, and outcome reporting, the available data provide a generally consistent signal that complete revascularization may reduce recurrent ischemia, reinfarction, and repeat revascularization, with a possible long-term survival benefit in selected patients. Therefore, the conclusions of this review should be interpreted as supportive of individualized, risk-based decision-making rather than as definitive evidence for a universal strategy.

The timing of non-culprit PCI remains an unresolved issue. Immediate revascularization during the index procedure may limit early recurrent ischemic events, while staged approaches may offer advantages in terms of patient stability and procedural safety. Current evidence does not establish a single best strategy, and the decision is often guided by operator judgment, anatomical considerations, and clinical presentation.

Given the substantial heterogeneity of NSTEMI patients with multivessel disease, a single broadly applicable treatment strategy is difficult to define. Age, frailty, diabetes, chronic kidney disease, bleeding risk, time from symptom onset, hemodynamic status, and lesion complexity may all modify the net benefit of complete revascularization. Therefore, the available evidence should be translated into clinical practice as a risk-adapted framework rather than a universal algorithm. Complete PCI may be appropriate in stable patients with significant residual ischemic burden and acceptable procedural risk, whereas culprit-only or staged approaches may be preferable in frail, comorbid, renally impaired, or anatomically complex patients. Treatment decisions should therefore be individualized, balancing ischemic protection against procedural safety.

Although NSTEMI management requires individualized decision-making, previous clinical evidence remains highly relevant in daily practice. Data from randomized trials, registries, observational studies, and accumulated clinical experience provide a framework for estimating the potential benefits and risks of different revascularization strategies. These data help identify general patterns, such as the association between complete revascularization and reduced recurrent ischemia or repeat revascularization, while also highlighting subgroups in whom procedural risk may outweigh benefit. Therefore, prior evidence should be used as a decision-support tool rather than a rigid algorithm. In clinical practice, the optimal strategy should integrate the best available evidence with patient-specific factors, including age, frailty, comorbidities, renal function, bleeding risk, clinical stability, lesion complexity, and patient preference.

Despite these insights, important gaps persist. Robust randomized trials specifically targeting NSTEMI patients with multivessel disease are scarce, and most recommendations are extrapolated from STEMI or mixed ACS cohorts. Moreover, long-term data on patient-centered outcomes, quality of life, and functional recovery are limited. The role of comorbid conditions and frailty in modifying outcomes also requires further investigation.

In summary, while the balance of evidence favors complete revascularization in NSTEMI with multivessel disease, decisions regarding extent and timing should remain individualized, integrating ischemic risk, procedural safety, and patient comorbidity. Until high-quality randomized data become available, the optimal strategy will continue to rely on a tailored, patient-centered approach informed by clinical judgment and multidisciplinary discussion.

## Figures and Tables

**Table 1:** Observational studies on complete vs. incomplete PCI revascularization in NSTEMI with multivessel disease.

Study/Year	Patients (*n*)	Strategy Compared	Major Exclusion Criteria/Follow-Up	Findings	Ref.
BLEE-Macs Registry/2017	ACS (*n* = 4520) ~67.7% STEMI, ~32.3% NSTEMI	Complete vs. incomplete revascularization	No major exclusion criteria/ 1 year	Primary endpoint (NSTEMI): lower 1-year death among CR group; The secondary endpoints (NSTEMI): lower in-hospital reinfarction, in-hospital heart failure, myocardial infarction, bleeding, and MACE at 1 year	[45]
Rathod et al./2018	NSTEMI (n = 37.491) NSTEMI + MVD (*n* = 21.857)	Complete vs. culprit-only PCI during index hospitalization	Cardiogenic shock; previous CABG; CTO/ 4.6 years	In-hospital MACE similar—slightly higher early mortality in CR but fewer repeat PCI.; Long-term follow-up—CR with lower all-cause mortality compared to CO	[48]
Correia et al./2018	NSTEMI + MVD (*n* = 202)	Complete revascularization (one-staged or second-staged) vs. incomplete revascularization	Previous CABG/ 1543 ± 545 days	Similar in-hospital adverse events. CR with lower rates of reinfarction, unplanned revascularization, unplanned PCI and the composite of death/reinfarction/unplanned revascularization compared with culprit-only PCI	[49]
Li T et al./2021	NSTE-ACS and MV-CAD (*n* = 3338)	Immediate multivessel vs. single-vessel PCI	STEMI, SCAD, no significant lesions; single vessel disease prior CABG, GFR < 30 mL/min/1.73 m^2^, cardiogenic shock/ 2 years	No significant difference of MACCE.	[52]
Pustjens et al./2022	NSTE-ACS + MVD (*n* = 10,507)	Multivessel-PCI vs. culprit-only PCI during index hospitalization	Out-of-hospital cardiac arrest, cardiogenic shock, previous CABG, CTO/ Up to 4 years	Primary outcome: all-cause mortality; (long-term): no difference. Secondary outcomes: early MI ≤30 d similar; reinterventions lower with MV-PCI; TVR at 1 year: Lower with MV-PCI but NS after matching; Urgent CABG <1 day: Lower with MV-PCI but NS after matching	[53]
Pandit et al./2022	NSTEMI + MVD (*n* = 60)	Complete revascularization (majority single-session) vs. target-vessel revascularization	>90% stenosis in ≥2 epicardial arteries; cardiogenic shock/ Outcomes assessed at 1 month and 6 months	Primary endpoints (all-cause death, non-fatal MI, ischemia-driven revascularization): no significant differences between CR and TVR at 1 or 6 months; Secondary endpoints: heart-failure hospitalizations and angina episodes were significantly lower with CR	[54]
e-Ultimaster Subgroup analysis/2025	NSTEMI + MVD *n* = 4353 (from 37.198 ACS in total)	Complete vs. incomplete revascularization during index hospitalization	No major exclusion criteria/ 1 year	CR associated lower POCE, mortality, combined risk of cardiac death and myocardial infarction and required fewer repeat revascularizations, particularly in non-target vessels; no adjusted difference in TLF.	[55]
Kim YH et al./2020	NSTEMI + MVD *n* = 4588	CO-PCI vs. MV-PCI; CR vs. IR during index hospitalization with new generation DES	Single vessel disease; MV-PCI performed staged after hospital discharge/2 years	In terms of MACEs and stent thrombosis: no significant differences in cumulative incidences across CO-PCI vs. MV-PCI, CR vs. IR, or CR vs. CO-PCI; Non-TVR was higher when revascularization was less complete.	[56]

ACS—Acute coronary syndrome; NSTEMI—Non-ST-elevation myocardial infarction; MVD—Multi-vessel disease; NSTE-ACS—Non-ST-segment elevation acute coronary syndrome; CAD—coronary artery disease; MVD—multivessel coronary artery disease; PCI—Percutaneous coronary intervention; CTO—Chronic Total Occlusion; POCE—Patient Oriented Composite Endpoint (all death, any myocardial infarction and any revascularization); TLF—Target Lesion Failure; CO—PCI —culprit only percutaneous coronary intervention; CR—complete revascularization; IR— incomplete revascularization; DES—drug-eluting stent; CABG—coronary artery bypass graft; STEMI—ST-segment elevation myocardial infarction; SCAD—stable coronary artery disease; GFR—glomerular filtration rate; MACE—major adverse cardiovascular events; MACCE—major adverse cardiac and cerebrovascular events; NS—not significant TVR—target vessel revascularization; MV-PCI—multivessel percutaneous coronary intervention; BLEE-Macs—Bleeding complications in a Multicenter registry of patients discharged with diagnosis of Acute Coronary Syndrome.

**Table 2:** Randomized controlled trials regarding the optimal timing of revascularization by PCI among patients with NSTEMI/ACS and MVD.

Study/Year	Patients (*n*)	Strategy Compared	Major Exclusion Criteria	Key Findings	Ref.
SMILE trial 2016	NSTEMI (*n* = 584)	Single-stage CR vs. multistage CR (index hospitalization)	Cardiogenic shock; prior CABG/surgical candidates; SYNTAX score>32; CTO; severe valvular heart disease; GFR <60 mL/min/1.73 m^2^	Primary endpoints: Single-stage CR ↓ MACCE at 1 year; faster troponin decline; ↓ recurrent ischemia; no ↑ MI, stroke, CIN, bleeding.	[60]
BIOVASC Substudy/2024	NSTE-ACS (*n* = 917) NSTEMI (*n* = 790)	ICR vs. SCR (within 6 weeks from index procedure)	Absence of a clear culprit; previous coronary artery bypass grafting; cardiogenic shock; CTO.	Primary composite endpoint NS; Secondary endpoints: ICR reduces MI and repeat PCI without added risk (early <30days strongest benefit); no ↑ bleeding or CIN.	[61]
2-year results of the BIOVASC study/2025	ACS (*n* = 1525) NSTE-ACS (*n* = 917)	ICR vs. SCR	Same as BIOVASC	Primary composite outcome NS; Secondary outcomes: No sustained advantage of ICR; late catch-up of adverse events in NSTEMI, mainly repeat PCI; both strategies safe	[63]

SMILE trial—Impact of Different Treatment in Multivessel Non-ST-Elevation Myocardial Infarction Patients: One-Stage Versus Multistaged Percutaneous Coronary Intervention; BIOVASC trial—Immediate vs. Staged Complete Revascularization in Patients With Acute Coronary Syndrome and Multivessel Disease; NSTEMI—Non-ST-Segment Elevation Myocardial Infarction; NSTE-ACS—Non-ST-Segment Elevation Acute Coronary Syndrome; ACS—acute coronary syndrome; ICR—immediate complete revascularization; CR—complete revascularization; SCR—staged complete revascularization; CABG—Coronary Artery Bypass Grafting; SYNTAX score—Synergy Between PCI With Taxus and Cardiac Surgery score; PCI—Percutaneous Coronary Intervention; ICR—Immediate Complete Revascularization; MACCE—Major Adverse Cardiac and Cerebrovascular Events; MI—myocardial infarction; CIN—Contrast Induced Nephropathy; CTO—Chronic Total Occlusion; GFR—Glomerular Filtration Rate; NS—nonsignificant; ↓—reduction/decrease; ↑— increase.

**Table 3:** Observational studies regarding the optimal timing of revascularization by PCI among patients with NSTEMI and MVD.

Study	Year	Patients (*n*)	Strategy Compared	Major Exclusion Criteria	Follow-Up	Key Findings	Ref.
C. Rao et al.	2025	NSTEMI + MVD (*n* = 2460)	Complete vs. incomplete PCI; Early vs. delayed multistaged complete revascularization	No MVD, in-hospital deaths, prior CABG	Median follow-up 528 days	Lower risk of MACCEs and MACE in CR group; Early multistage CR significantly better than delayed multistage CR.	[65]
Alici et al.	2021	NSTEMI + MVD (*n* = 298)	Single vs. multistage PCI	Prior CABG SYNTAX score > 32 LVEF < 40% Severe VHD, History of CPR, Malignancy, GFR < 30 mL/min/1.73 m^2^, chronic liver disease, candidates for cardiac surgery	2 years	In-hospital mortality higher with single-stage PCI (NS after multivariable analysis); 2-year mortality similar between groups; Independent predictors of mortality: low hemoglobin, no-reflow and lack of post-dilatation	[66]
FRAME-AMI post hoc analysis	2024	AMI + MVD (*n* = 549) NSTEMI + MVD (*n* = 295)	Immediate vs. staged PCI	Cardiogenic shock; LVEF < 25%; GFR < 30 mL/min/1.73 m^2^; prior/candidates CABG, CTO, severe VHD, life expectancy <1y.		Primary composite outcome: NS—in NSTEMI patients’ outcomes were nearly identical between strategies; Secondary outcomes: All-cause mortality, MI, stroke, stent thrombosis—NS; unplanned revascularization: NS, in NSTEMI patients a late “catch-up” of repeat revascularization was observed	[67]
Wang et al.	2022	NSTEMI + MVD *n* = 943	Culprit only PCI, Immediate MV-PCI and out-of-hospital staged MV-PCI	Cardiogenic shock; failed PCI; death within 2 months after discharge; staged MV-PCI during the primary admission/ staged PCI beyond sixty days; prior CABG or within 60 days after primary admission.		Multivessel PCI better than culprit-only; staged PCI → lowest MACE & mortality.	[68]
Demirkıran et al.		NSTEMI+ MVD *n* = 316	ICR vs. SCR (SCR1 < 24h and SCR 2 > 24h) vs. NCR	UA; STEMI; history of prior PCI, CABG, stroke; GFR < 50 mL/min/1.72 m^2^; Killip class >2; LVEF <40%	Median follow-up 63 ± 28 months, with a maximum follow-up period of 120 months	ICR showed the best outcomes; SCR overall had higher events, but when completed ≤24h, outcomes were similar to ICR. >24h staging was much worse; NCR had the poorest outcomes, close to delayed SCR.	[69]

CABG—coronary artery by-pass grafting; PCI—percutaneous coronary intervention; VHD—valvular heart disease; GFR—glomerular filtration rate; CPR—cardiopulmonary resuscitation; SYNTAX score—SYNergy between Percutaneous Coronary Intervention with Taxus and Cardiac Surgery; NS—non-significant; NSTEMI—Non-ST-Segment Elevation Myocardial Infarction; STEMI—ST-Segment Elevation Myocardial Infarction; CTO—chronic total occlusion; MVD—multivessel disease; AMI—acute myocardial infarction; LVEF—left ventricular ejection fraction; ICR—immediate complete revascularization; SCR—staged complete revascularization; NCR—non-complete revascularization; UA—unstable angina; MV-PCI—multivessel percutaneous coronary intervention; MACE—Major Adverse Cardiac Events; CR—complete revascularization; MACCE—major adverse cardiac and cerebrovascular events; MI—myocardial infarction.

## Data Availability

No new data were created or analyzed in this study. Data sharing is not applicable to this article.

## Abbreviations

The following abbreviations are used in this manuscript:

ACS	Acute Coronary Syndrome
AHA/ACC	American Heart Association/American College of Cardiology
AMI	Acute Myocardial Infarction
BLEEMACS	Bleeding Complications In A Multicenter Registry Of Patients Discharged With Diagnosis of Acute Coronary Syndrome
CABG	Coronary Artery Bypass Grafting
CAD	Coronary Artery Disease
CI	Confidence Interval
CKD	Chronic Kidney Disease
CO	Culprit-Only
CR	Complete Revascularization
CTO	Chronic Total Occlusion
DAPT	Dual Antiplatelet Therapy
DES	Drug-Eluting Stents
ESC	European Society of Cardiology
GRACE	Global Registry of Acute Coronary Events
HEART	History, Electrocardiogram, Age, Risk Factors and Troponin
HR	Hazard Ratio
ICR	Immediate Complete Revascularization
IR	Incomplete Revascularization
IRA	Infarct Related Artery
LAD	Left Anterior Descending Coronary Artery
LCx	Left Circumflex Coronary Artery
LM	Left Main Coronary Artery
LVEF	Left Ventricular Ejection Fraction
MACCE	Major Adverse Cardiac and Cerebrovascular Events
MACE	Major Adverse Cardiac Events
MeSH	Medical Subject Heading
MI	Myocardial Infarction
MS	Multi-stage
MVD	Multivessel Coronary Artery Disease
MV	Multi-vessel
NCR	Non-Complete Revascularization
NSTE-ACS	Non-ST-Elevation Acute Coronary Syndrome
NSTEMI	Non-ST-Segment Elevation Myocardial Infarction
PCI	Percutaneous Coronary Intervention
POCE	Patient-Oriented Composite Endpoint
RCA	Right Coronary Artery
SCR	Staged Complete Revascularization
SS	Single-Stage
STEMI	ST-Segment Elevation Myocardial Infarction
SYNTAX	Synergy Between PCI With Taxus and Cardiac Surgery
TIMI	Thrombolysis In Myocardial Infarction
UKGRIS	UK GRACE Risk Intervention Study
